# From vision loss to paralysis: a striking case of Wernicke’s encephalopathy in pregnancy

**DOI:** 10.1097/MS9.0000000000004117

**Published:** 2025-10-16

**Authors:** Fatima Ez-Zahra Mabrouki, Sanae El Hasnaoui, Samah Yousfi, Yassine Mebrouk

**Affiliations:** a Department of Neurology, Mohammed VI University Hospital, Oujda, Morocco; b Faculty of Medicine and Pharmacy, Mohamed First University, Oujda, Morocco

**Keywords:** hyperemesis gravidarum, hypokalemic paralysis, ophthalmologic manifestation, thiamine deficiency, Wernicke’s encephalopathy

## Abstract

**Introduction:**

Wernicke’s encephalopathy (WE) is a neurological emergency caused by thiamine deficiency, most often associated with chronic alcoholism, but it can also occur in non-alcoholic settings such as hyperemesis gravidarum.

**Case presentation:**

We report the case of a 37-year-old pregnant woman with intractable vomiting who developed blurred vision, ophthalmoplegia, and subsequently acute flaccid paraparesis. Brain MRI showed typical lesions of WE, and laboratory tests revealed profound hypokalemia. Clinical improvement followed intravenous thiamine and potassium supplementation.

**Clinical discussion:**

This case illustrates the dual neurological complications of hyperemesis gravidarum: Wernicke’s encephalopathy and hypokalemic paralysis, both resulting from severe nutritional and electrolyte depletion.

**Conclusion:**

Hyperemesis gravidarum can cause both thiamine deficiency and severe hypokalemia. Early recognition and combined correction are essential to prevent permanent neurological sequelae.

## Introduction

Wernicke’s encephalopathy (WE) is a rare but severe neuropsychiatric syndrome caused by thiamine (vitamin B1) deficiency. Although classically associated with chronic alcoholism, it can occur in any context of prolonged malnutrition or vomiting, including hyperemesis gravidarum^[1]^. The clinical triad-encephalopathy, ophthalmoplegia, and ataxia-is present in only a minority of cases, often delaying diagnosis and treatment^[2]^. We present the case of a young woman with hyperemesis gravidarum who developed Wernicke’s encephalopathy with a distinctive ophthalmological presentation, associated with acute hypokalemic flaccid paraparesis. This case illustrates the importance of recognizing the neurological complications of nutritional deficiencies during pregnancy.

## Case presentation

**Figure 1.**
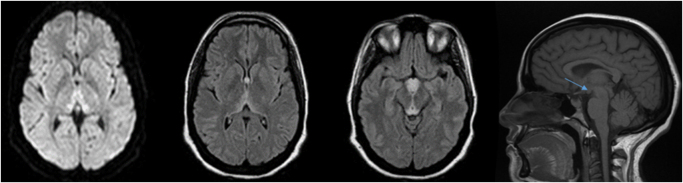
Brain MRI (axial and sagittal views, Diffusion, FLAIR and T1 sequences) showing bilateral hyperintensities in the medial thalami, mammillary bodies, and periaqueductal region.

**Figure 2.**
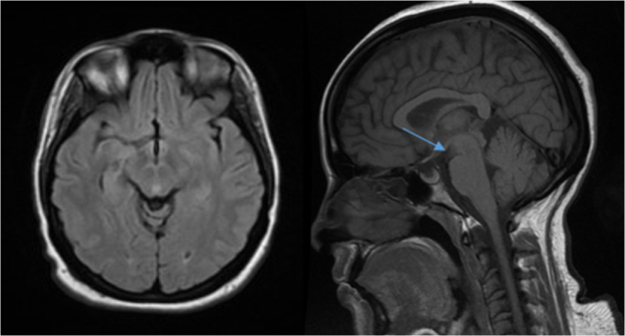
Follow-up MRI showing resolution of FLAIR hyperintensities and mammillary body atrophy.

A 37-year-old woman at 16 weeks of gestation was admitted to the emergency department with persistent vomiting lasting 2 weeks. She reported the recent onset of intense headaches, blurred vision, and horizontal diplopia. Neurological examination revealed bilateral sixth nerve palsy without motor or sensory deficits. Fundoscopic examination showed bilateral grade II papilledema and a peripapillary hemorrhage. Initial brain MRI, including MR angiography sequences, was unremarkable. Lumbar puncture and extensive laboratory tests including metabolic, infectious, and autoimmune panels were within normal limits. Over the following days, the patient’s condition deteriorated with the onset of confusion and a flaccid, areflexic paraparesis. Follow-up brain MRI revealed bilateral symmetrical FLAIR hyperintensities involving the periaqueductal gray matter, mammillary bodies, optic chiasm, the periventricular region of the third ventricle, and both medial thalami (Fig. 1), consistent with WE. Electroneuromyography and spinal MRI were normal. Severe hypokalemia (K^+^ < 2.5 mmol/L) was identified, supporting the diagnosis of hypokalemic paralysis. The patient was treated with intravenous thiamine and potassium chloride, resulting in gradual clinical improvement. Follow-up brain MRI showed regression of the FLAIR hyperintensities and mammillary body atrophy (Fig. 2).

**HIGHLIGHTS**

- Hyperemesis gravidarum can lead to serious complications, including Wernicke’s encephalopathy (WE) and hypokalemic paralysis, as a result of vitamin and electrolyte depletion.
- WE may initially present with blurred vision and ophthalmologic signs, including retinal involvement, even before classic features appear.
- Profound hypokalemia can cause flaccid paralysis, even in the absence of abnormalities on spinal MRI or nerve conduction studies.
- MRI is essential for early diagnosis of WE, typically revealing bilateral symmetrical lesions in the thalami, mammillary bodies, and periaqueductal gray matter.
- Early combined treatment with intravenous thiamine and potassium is critical to avoid irreversible neurological damage and to ensure favorable outcomes.

## Discussion

Hyperemesis gravidarum is defined as intractable vomiting during the first trimester of pregnancy, leading to dehydration, weight loss, electrolyte imbalances, and in severe cases, nutritional deficiencies including thiamine deficiency^[1]^. Although rare, WE is a known complication and constitutes a medical emergency. Without prompt recognition and treatment, it can lead to coma, persistent neurologic sequelae, or death.

Thiamine is a coenzyme essential to carbohydrate metabolism. Its deficiency primarily affects brain regions with high metabolic demand, such as the medial thalami, mammillary bodies, and periaqueductal gray matter. MRI findings in WE typically show symmetrical hyperintensities in these regions on T2/FLAIR sequences^[3]^. The clinical triad of confusion, ophthalmoplegia, and ataxia is reported in only 16.5% of cases^[2]^. In our patient, the ophthalmologic presentation was atypical, with bilateral papilledema and a peripapillary hemorrhage, suggestive of retinal ischemia due to Thiamine deficiency. Retinal manifestations in WE are rarely reported, though isolated cases describe papilledema and flame-shaped retinal hemorrhages^[3]^.

A striking feature of this case was the concomitant development of flaccid paraparesis associated with profound hypokalemia. Although hypokalemia is common in hyperemesis gravidarum, paralytic forms are rare. In a study of 406 patients with serum potassium levels below 3 mmol/L, paralysis was reported in only 14 cases^[4]^. Typically, hypokalemic paralysis presents as an ascending, symmetric flaccid quadriparesis with reduced or absent deep tendon reflexes, as seen in our patient.

The coexistence of WE and hypokalemic paralysis in this case reflects the dual impact of severe vomiting during pregnancy. Treatment requires urgent intravenous thiamine supplementation, followed by oral maintenance, as well as correction of electrolyte imbalances to prevent permanent neurological sequelae or maternal-fetal complications.

## Conclusion

This case underlines the need for heightened clinical awareness of WE and hypokalemic paralysis in the setting of hyperemesis gravidarum. The unusual ophthalmologic onset with retinal involvement, along with the rare neuromuscular complication, highlights the broad clinical spectrum of both thiamine and potassium deficiencies. Early neuroimaging and laboratory evaluation, combined with prompt administration of thiamine and potassium, are essential for a favorable outcome.

## Take-away lessons

- Hyperemesis gravidarum can lead to serious complications, including WE and hypokalemic paralysis, as a result of vitamin and electrolyte depletion.
- WE may initially present with blurred vision and ophthalmologic signs, including retinal involvement, even before classic features appear.
- Profound hypokalemia can cause flaccid paralysis, even in the absence of abnormalities on spinal MRI or nerve conduction studies.
- MRI is essential for early diagnosis of WE, typically revealing bilateral symmetrical lesions in the thalami, mammillary bodies, and periaqueductal gray matter.
- Early combined treatment with intravenous thiamine and potassium is critical to avoid irreversible neurological damage and to ensure favorable outcomes.

## Data Availability

Available upon reasonable request.
